# THE WIDOWHOOD EFFECT IN COMPLEX SERIOUS ILLNESS: THE IMPACT OF SPOUSAL DEATH ON MORTALITY IN DEMENTIA

**DOI:** 10.1093/geroni/igac059.1369

**Published:** 2022-12-20

**Authors:** Rebecca Rodin, Alex Smith, Edie Espejo, W John Boscardin, Siqi Gan, Lauren Hunt, Katherine Ornstein, Sean Morrison

**Affiliations:** Icahn School of Medicine at Mount Sinai, New York City, New York, United States; University of California, San Francisco, San Francisco, California, United States; University of California, San Francisco, San Francisco, California, United States; University of California, San Francisco, San Francisco, California, United States; University of California, San Francisco, San Francisco, California, United States; University of California, San Francisco, San Francisco, California, United States; Johns Hopkins, University, Baltimore, Maryland, United States; Icahn School of Medicine at Mount Sinai, New York City, New York, United States

## Abstract

Numerous studies suggest that there is an association between widowhood and mortality. This “widowhood effect” may be heightened in patients with dementia, who have high support needs and for whom spouses typically provide extensive caregiving support. Yet there are limited data on widowhood and mortality that account for dementia status. To determine the relative mortality risk of widowhood among those with and without dementia, we conducted a retrospective cohort study among community-dwelling, married/partnered persons, ≥65 years, enrolled in the Health and Retirement Study, 2000-2018. Among the 12,308 persons (n=390 with dementia), widowhood was not associated with increased mortality, after adjusting for age and dementia status, in men or women (adjusted HR 1.04; 95%C.I.(0.95-1.13); HR 0.96; 95%C.I.(0.87-1.95), respectively). These findings suggest that dementia, age, or other unmeasured confounding variables may account for the previous finding of increased mortality following spousal death. Further research is needed to confirm these findings in diverse populations.

